# Blood pressure lowering in pregnancy

**DOI:** 10.1111/jch.14247

**Published:** 2021-03-30

**Authors:** Piotr Sobieraj

**Affiliations:** ^1^ Department of Internal Medicine, Hypertension and Vascular Diseases Medical University of Warsaw Warsaw Poland

Liu et al^1^ demonstrated the effect of various states of hypertension and other factors on low birth weight (LBW) and small for gestational age (SGA) in pregnancy. In the large prospective study, the authors showed how strongly the diagnosis of hypertension in pregnancy and pre‐eclampsia affects the risk of LBW and SGA. The authors conclude that their results may be a premise for preventive measures to identify risk factors for an unfavorable pregnancy outcome related to hypertension.

However, according to the current state of knowledge, the possibilities of preventing the negative effects of high blood pressure in pregnancy are limited. In the randomized Control of Hypertension in Pregnancy **S**tudy (CHIPS), which aimed to compare the reduction in diastolic blood pressure to tight control <85 mmHg and less‐tight control <100 mmHg, no difference according to SGA (defined as birth weight <10th percentile or <3rd percentile) was found.^2^ There were also no differences in terms of risk of pregnancy loss, high‐level neonatal care, and maternal complications. Merely, it was shown that tight control was associated with the lower incidence of severe maternal hypertension in comparison with less‐tight control.

Regarding Liu's results, it is interesting how blood pressure lowering would affect LBW/SGA risk. Likewise, since the target blood pressure in pregnancy is unknown, it is extremely important to understand the relationship between achieved blood pressure and pregnancy outcomes. Unfortunately, no blood pressure values were reported.

According to the position paper on the peripartum management of hypertension by the European Society of Cardiology Council on Hypertension and the European Society of Hypertension, these issues should be addressed to researchers in the field of hypertension in pregnancy as they represent significant gaps in knowledge.^3^

## CONFLICT OF INTEREST

The author declares that there is no conflict of interest.

## AUTHOR CONTRIBUTIONS

Piotr Sobieraj contributed to the conception of the manuscript, drafting the review paper, and final approval of the version to be published.
